# The effect of methods used in the management of maternal obesity on pregnancy and birth outcomes: a systematic review with meta-analysis

**DOI:** 10.1038/s41366-025-01748-y

**Published:** 2025-03-26

**Authors:** Döndü Kurnaz, Zekiye Karaçam

**Affiliations:** 1 Marmara University, Faculty of Health Sciences, Division of Midwifery, Istanbul, Turkey; 2 Aydın Adnan Menderes University, Faculty of Health Sciences, Division of Midwifery, Aydın, Turkey

**Keywords:** Lifestyle modification, Epidemiology

## Abstract

**Aim:**

This study was conducted to determine the effects of the methods used in the management of maternal obesity on pregnancy and birth outcomes.

**Materials and methods:**

This study was conducted following the PRISMA Statement. The articles to be used in the meta-analysis were searched in PubMed, National Thesis Center, DergiPark, MEDLINE, Cochrane Library and EBSCO search engines in October 2021 and updated in September 2023. The methodological qualities of the studies were evaluated using ROB2. The data were synthesized using meta-analysis, and the GRADE approach was used to rate the certainty of the evidence and the strength of the recommendations. Twenty-one studies published between 2013 and 2021 were included in the study. The total sample size of the studies was 7695.

**Results:**

Weight management interventions significantly reduced weight gain during pregnancy (*p* < 0.001) and birth weight (*p* < 0.01). Did not affect other adverse pregnancy outcomes included in the synthesis (*p* > 0.05). The subgroup analyses showed that the method of handing out brochures resulted in lower levels of birth weight (*p* < 0.01) and weight gained during pregnancy (*p* < 0.001); the use of metformin was associated with a significant drop in admissions to the neonatal intensive care unit (*p* < 0.01); the method of exercise was associated with lower in gestational diabetes (*p* < 0.001), weight gained during pregnancy (*p* < 0.001), birth weight (*p* = 0.01) and large-for-gestational-age baby birth (*p* < 0.05), while and the combination of diet and exercise significantly reduced weight gained during pregnancy (*p* = 0.001). The certainty of evidence assessed using GRADE for all 15 critical outcomes was high 15 outcomes.

**Conclusion:**

The study revealed that methods used in the treatment of maternal obesity may reduce some negative maternal and newborn outcomes, but it is more important to start pregnancy with an ideal weight.

## Introduction

The World Health Organization (WHO) regards obesity as an epidemic with serious social and psychological consequences, defining it as “excessive fat accumulation in the body that can impair health, in addition to its effects on physical health” [1, 2]. The prevalence of obesity among women of reproductive age increases maternal obesity rates, which have short- and long-term negative consequences for maternal and infant health [3]. The prevalence of maternal obesity varies by country and country income levels [4–10]. Obesity can affect the whole of a woman’s life during the periods of pre-pregnancy, pregnancy, birth, and postpartum, posing a fundamental problem for mother-baby health [11, 12]. Maternal obesity is associated with a higher risk of pregnancy and poor perinatal outcomes. It is also known that pregnant women with obesity may be exposed to discrimination and stigmatization during this period when they should be cheerful and happy [13], and the rates of antepartum and postpartum depression are higher among these pregnant women [12]. Therefore, women with obesity should be considered at elevated risk and should be carefully managed [3]. The WHO highlights few specific policies that are thought to be effective in reducing obesity; these policies include pre-pregnancy and gestational care along with maternal obesity management [14]. It has also been reported that the management of maternal obesity is beneficial to neonatal health, in addition to the health of mothers with overweight and obesity [15]. Moreover, it was reported in a study that women are motivated to adopt healthy behavioral changes and lifestyles because they believe that by doing so, they will protect the health of their children during pregnancy, and the obesity cycle that can last for generations can be broken with lifestyle programs [16]. To reduce the burden of maternal obesity, women with obesity need support in losing weight in the pre-pregnancy period and in minimizing weight gain during pregnancy. Health professionals are effective in managing maternal obesity and can break the obesity cycle. To provide this support and plan quality care, evidence comprising up-to-date data is needed. It is reported in a Cochrane review published in 2013 on prenatal interventions for weight loss to improve pregnancy outcomes in women with obesity, it is reported that there are no studies on weight management interventions to be applied in pregnancy and that therefore more studies are needed to investigate potential benefits and risks [17]. There have been randomized controlled trials (RCT) based on strong evidence that have been published in the literature after this date in used in the management of obesity during pregnancy are employed. In these studies, the effectiveness of interventions such as diet, physical activity, metformin use, and lifestyle changes were analyzed.

When the current meta-analysis studies were examined, pregnant women with overweight and higher body weight were studied together and/or only one method of intervention was examined [18, 19]. This study was initiated upon the observation that there was a need for high levels of evidence-based knowledge that would include the results of current RCTs and all intervention methods used. In addition, it is important to update data in today’s world, especially in view of the increase in communication tools and accessibility, the many changes in lifestyles and nutritional habits that are related to maternal obesity. It was for these reasons that this systematic review and meta-analysis aimed to seek out the strong evidence-based results of current studies that were performed only on pregnant women with obesity and that used all the methods mentioned. It is believed that the obtained information can contribute to reducing the negative pregnancy outcomes associated with maternal obesity and breaking the obesity cycle. This systematic review and meta-analysis aimed to determine the effectiveness of approaches for the management of maternal obesity based on previous primary studies. This research aimed to answer the following question: What are the effects of the methods used in the management of maternal obesity on mother-infant health?

## Materials and methods

The “PRISMA Statement: Preferred Reporting Items” checklist was followed in creating the study protocol of this systematic review and meta-analysis and in writing the article [20]. The study protocol was registered in PROSPERO under the registration number CRD42021226482 dated 22.01.2021. In this systematic review, the scans, the selection of the studies, the first author, and a volunteer researcher independently performed data extraction and quality assessment.

### Conformity criteria

Studies eligible for this systematic review were determined according to the following PICOS criteria: Patient: Pregnant women with obesity and their babies. Intervention: Interventions used for weight management in pregnancy such as exercise, diet, metformin use, handing out brochures. Comparison: pregnant women with obesity and their infants who did not use methods for managing maternal obesity. Outcomes: Maternal and neonatal outcomes reported in the studies. Study design: RCTs published in Turkish and English were included in the study.

### Exclusion criteria

Studies whose research findings were not suitable for meta-analysis, that displayed that had small sample sizes (*n* = 10), those that were conducted with groups with chronic diseases in addition to obesity, and where Body Mass Index (BMI) was not evaluated according to the WHO criteria, studies other than RCT and were not included in this study. Studies with inappropriate statistical analyses, pilot studies, studies without full-text access, studies with different outcome outcomes, and studies with different samples were not included in the synthesis of this systematic review.

### Search strategy

The scans were independently conducted by the first investigator and a volunteer researcher between September and October 2021 and were updated in September 2023. The PubMed, MEDLINE, Cochrane Library, EBSCO, Web of Science, National Thesis Center, and DergiPark. The words and phrases Obesity* AND (pregnancy* OR “babies health” OR “maternal Health” OR management) were used in the scans. For additional searches, studies included in the systematic review and reference lists of previous systematic reviews were checked.

### Selection of articles

The studies included in this systematic review were determined by removing repetitive studies in the scans and selecting them according to title, abstract, and full text. Two researchers independently selected the articles, and when there was a difference of opinion about any study, a consensus was reached through discussion with the second author.

### Evaluation of the methodological quality of the studies

The methodological quality of the studies was evaluated using RoB2, the Cochrane bias risk tool developed for RCTs [21]. In addition, the GRADE program proposed by the Cochrane working group was used to evaluate the certainty of the evidence for all critical outcomes identified in the research questions and to rate the strength of the recommendations [22].

### Data extraction

The data extraction tool developed by the Joanna Briggs Institute [2020] was used to obtain the research data, with suitable changes made for the study [23]. Data extraction was performed independently by two researchers and was converted into a single text in a joint session.

### Synthesis of the data

Review Manager 5.4.1 was used for the meta-analysis. The heterogeneity test, Cochran’s Q test and Higgins I² were used for the evaluation; a rate of >50% for I² was accepted as indicating significant heterogeneity. Random Effects results were used if I² was more than 50%, and Fixed Effects results were used if I2 was 50%. The Odds Ratio was calculated for categorical variables and the Mean Difference for continuous variables. All tests were calculated on a two-tailed basis, and a *p* value of less than 0.05 was set to indicate statistical significance. In addition, subgroup analysis was performed for sensitivity analysis according to the type of intervention [21].

## Results

### Searching results

**Fig. 1:**
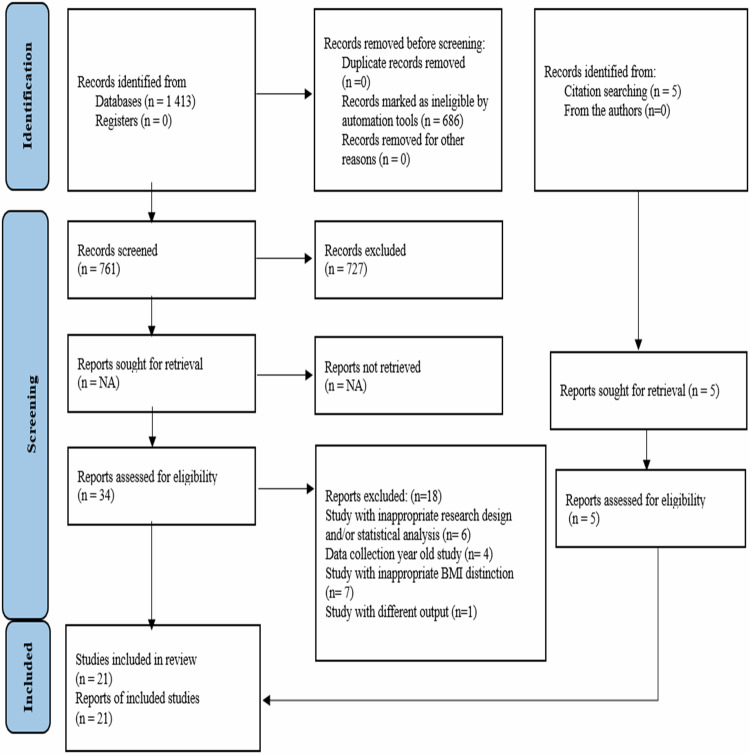
PRISMA flowdiagram of the search process.

In this study, 1413 records were obtained from the scans made from the databases, and five records were obtained from the additional scans. As a result of the removal of repetitive records and analysis according to title and summary, 39 studies were selected to be examined in the full text. After examining the full texts of these studies according to the inclusion criteria, 21 studies were included in the meta-analysis (Fig. 1).

### Characteristics of studies and participants

All studies included in this systematic review were RCTs. In addition, three studies had two intervention groups, and the results of both interventions were analyzed [24–26]. It was found that the countries where the studies were conducted were England [26–32], Norway [33–35], Denmark [36–38], Belgium [24, 25], United States [39, 40], China [41], Italy [42], Brazil [43], and England & Scotland [44]. Studies were conducted between 2007 and 2017 and published between 2013 and 2021. The total sample size of the studies was 7695 (intervention group: 4070; control group: 3625). The mean BMI of the mothers in the study sample was at the lowest 33.09 ± 7.34, at the highest 39.00 ± 26.20, and the lowest mean age was 28.67 ± 3.68; the highest was 32.50 ± 4.91 (Supplementary-File Table 1).

### Features of the intervention

Of the studies included in this systematic review and meta-analysis, lifestyle interventions consisting of diet and exercise were used in 14 [24, 25, 28–32, 36–40, 42, 44], exercise in five [26, 33–35, 41], diet in one [26], brochure distribution in two [24, 25], and metformin in two studies [27, 43]. Interventions for women were initiated and applied during pregnancy. It was determined that the time the intervention was started in the studies was at gestational weeks 7–21; in five studies, the intervention was started at gestational weeks 15–19 [28–31, 44] (Supplementary-File Table 1).

### Results of the quality assessment of the studies

**Fig. 2:**
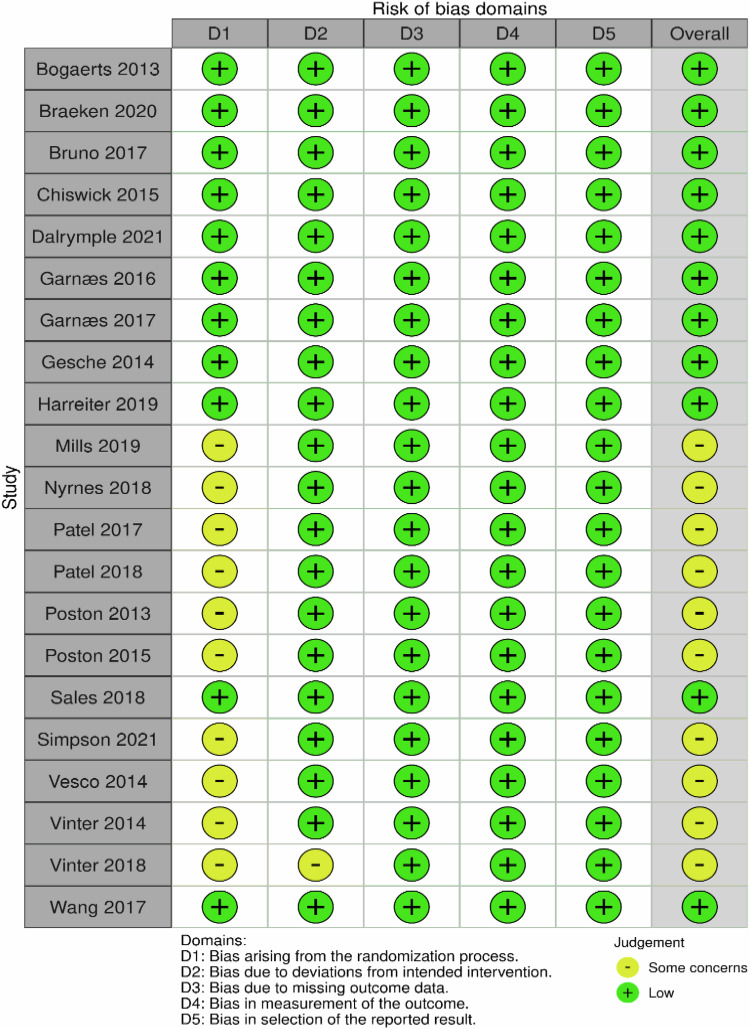
Risk of bias’ domains: RoB-2.

In the methodological quality evaluation of the studies included in the meta-analysis, low risk was determined in 11 studies [24–28, 33, 34, 36, 41–43], and moderate risk was determined in 10 studies [29–32, 35, 37–40, 44]. In studies at moderate risk, there was no information that any remote or centrally managed method was used to assign participants to interventions during the randomization process or that the separation process was controlled by an external unit or organization independent of the enrollment staff (Fig. 2).

### Meta-analysis results

In this meta-analysis, which was based on the findings of 21 RCTs, information on the effects of diet, exercise, diet-exercise, brochure distribution, and metformin used in the management of maternal obesity, data were revealed on a total of 31 outcome variables related to mother/infant health.

In the 13 studies reviewed [24, 27, 29–33, 37, 40–44], results were reported regarding the effect of applying the treatment to pregnant women with obesity on gestational diabetes. In the meta-analysis, interventions applied to pregnant women with obesity reduced the occurrence of gestational diabetes, but the finding was not statistically significant (*z* = 1.90, *p* = 0.06). In the subgroup analysis performed by intervention type, it was found that the exercise intervention statistically and significantly reduced the occurrence of gestational diabetes (*z* = 3.42, *p* = 0.0006), but the other methods were not effective (Table 1, Supplementary-File-Fig. 1).

**Table 1:** Findings on the effects of methods used in the management of maternal obesity on maternal health during pregnancy, childbirth, postpartum period and neonatal period.

Outcomes	Number of Studies	Intervention group Cases/Total Sample size	Control group Cases/Total Sample size	Odds Ratio/Mean Difference [%95 CI]	Heteregenity			Overall Impact
					Tau^2^	X^2^/df/p	I^2^	z/p
Outcomes in the pregnancy period								
Gestational diabetes mellitus	13	597/2902	654/2878	0.88 [0.78–1.00]	–	19.65/13/0.10	34	1.90/0.06
Gestational hypertension	6	60/685	67/693	0.92 [0.64–1.33]	–	11.61/6/0.07	48	0.45/0.65
Pre-eclampsi	7	69/1724	64/1739	1.10 [0.78–1.56]	–	3.88/7/0.79	0	0.54/0.59
Eclampsia	1	6/783	10/772	0.59 [0.21–1.63]	–	–	–	1.02/0.31
Abortion	2	25/1009	16/995	1.55 [0.82–2.93]	–	1.34/1/0.25	25	1.36/0.17
Gaining more weight than IOM recommendations during pregnancy	3	120/383	107/393	1.07[0.54–2.13]	0.24	5.74/2/0.06	65	0.20/0.84
Gestational weight gain	9	2206	2218	1.24 [−1.83 ^_^ −0.65]	0.55	32.47/9/0.0002	72	4.10 / < 0.0001
Outcomes in the intrapartum period								
Vaginal delivery	7	741/1457	787/1550	1.04 [0.90–1.20]	–	5.65/7/0.58	0	0.49/0.62
Instrumental delivery	7	161/1416	162/1495	1.03 [0.82–1.30]	.	5.30/7/0.62	0	0.26/0.80
Emergency cesarean delivery	5	193/1253	247/1357	0.89 [0.58–1.36]	0.15	13.16/5/0.02	62	0.54/0.59
Elective cesarean delivery	5	245/1253	242/1357	1.03 [0.71–1.50]	0.10	10.29/5/0.07	51	0.16/0.87
Caesarean delivery	11	587/1832	667/1928	0.89 [0.76–1.02]	–	10.32/11/0.50	0	1.66/0.10
Preterm delivery	7	31/1381	29/1388	1.10 [0.66–1.83]	–	6.44/6/0.38	7	0.36/0.72
Induction of labour	3	321/1013	339/993	0.90 [0.74–1.08]	–	2.28/3/0.52	0	1.13/0.26
Outcomes in the postpartum period								
Postpartum haemorrhage	2	149/1009	122/995	1.24 [0.96–1.61]	–	0.90/1/0.34	0	1.64/0.10
Perineal laceration	1	4/18	2/10	1.14 [0.17–7.69]	–	–	–	0.14/0.89
Outcomes in the neonatal period								
Birth weight [gr]	11	2372	2448	−51.84 [−85.16–17.83]	1682.10	151984.19/13 / < 0.00001	100	2.99/0.003
Large for gestational age	8	158/1881	192/1915	0.85 [0.54–1.33]	0.29	25.76/8/0.001	69	0.73/0.47
Small for gestational age	4	42/741	35/736	1.20 [0.76–1.90]	–	2.85/4/0.58	0	0.78/0.44
Macrosomia	9	254/1939	276/1951	0.90 [0.75–1.09]	–	13.47/9/0.14	33	1.09/0.28
Low birth weight	2	30/665	34/656	0.86 [0.52–1.43]	–	2.33/2/0.31	14	0.57/0.57
Congenital anomaly	2	12 /1009	14/995	0.84 [0.39–1.84]	–	0.00/1/0.95	0	0.43/0.67
Neonatal intensive care unit admission	5	89/1136	105/1143	0.86 [0.64–1.15]	–	7.41/4/012	46	1.02/0.31
Perinatal death	2	10/1009	14/995	0.70 [0.31–1.59]	–	0.10/1/0.76	0	0.85/0.39
Breastfeeding problems	2	219/592	311/620	0.62 [0.28–1.35]	0.29	11.09/1 / < 0.0009	91	1.21/0.22

In the meta-analysis, it was determined that the incidence of gestational hypertension was lower in the intervention group, but this result was not statistically significant (*z* = 0.45, *p* = 0.65). The subgroup analysis according to the type of intervention applied yielded similar results (Table 1, Supplementary-File-Fig. 2).

The meta-analysis showed that the interventions implemented did not affect the development of preeclampsia and eclampsia (respectively; *z* = 0.54, *p* = 0.59; *z* = 1.02, *p* = 0.31; Table 1, Supplementary-File-Fig. 3).

The meta-analysis showed that weight management interventions did not affect miscarriage (*z* = 1.36, *p* = 0.17). Similar results were obtained in the subgroup analysis according to the type of intervention (Table 1, Supplementary-File-Fig. 4).

This meta-analysis found that weight management interventions significantly reduced weight gain during pregnancy (*z* = 4.10, *P* < 0.0001). In the subgroup analysis based on the type of intervention, it was determined that the metformin intervention did not affect weight gain. However, weight gain during pregnancy decreased significantly in the groups that received leaflets, practiced diet exercise, and participated in an exercise intervention (*z* = 1.03, *p* = 0.30; *z* = 3.12, *p* = 0.002; *z* = 3.23, *p* = 0.001; *z* = 5.18, *p* < 0.00001, respectively). In addition, showed that the intervention did not statistically affect the number of pregnant women who gained excessive weight during pregnancy (*z* = 0.20, *p* = 0.84; Table 1, Supplementary-File-Fig. 5).

The meta-analysis showed that interventions did not affect spontaneous vaginal and instrumental delivery (respectively; *z* = 0.49, *p* = 0.62; *z* = 0.26, *p* = 0.80). The results of subgroup analyses according to the type of intervention applied were similar (Table 1, Supplementary-File-Fig. 6).

In the meta-analysis based, it was found applied in pregnancies with obesity did not affect the rates of elective cesarean, emergency cesarean (respectively; *z* = 0.16, *p* = 0.87; *z* = 0.54, *p* = 0.59; Table 1, Supplementary-File-Fig. 7), or cesarean section delivery (*z* = 1.66, *p* = 0.10). Subgroup analyses by intervention type also showed that the intervention didn’t affect cesarean delivery rate (Table 1, Supplementary-File-Fig. 8).

In the meta-analysis, it was found that the interventions did not have an effect on the use of labor induction (*z* = 1.13, *p* = 0.26). The subgroup analyses according to the type of intervention also showed similar results (Table 1, Supplementary-File-Fig. 9).

In the meta-analysis, the interventions did not have an impact on preterm births (*z* = 0.36, *p* = 0.72). Subgroup analyses according to the type of intervention also yielded similar results (Table 1, Supplementary-File-Fig. 10).

Researchers reported postpartum hemorrhage in two studies [27, 32] and third- to fourth degree perineal laceration in another study [34] in which weight management interventions were applied to pregnant women with higher body weight. The pooled results of these interventions did not exhibit statistically significant differences (respectively; *z* = 1.64, *p* = 0.10; *z* = 0.14, *p* = 0.89; Table 1, Supplementary-File-Fig. 11).

The meta-analysis showed that weight management interventions for pregnant women with higher body weight statistically and significantly resulted in reduced birth weight (*z* = 2.99, *p* = 0.003). In the subgroup analysis, it was determined that giving brochures and exercise interventions were effective (respectively; *z* = 1018.82, *p* < 0.00001; *z* = 46, *p* = 0.01) but diet exercise, diet, and metformin interventions had no impact in this respect (respectively; *z* = 0.83, *p* = 0.41; *z* = 0.32, *p* = 0.75; *z* = 0.02, *p* = 0.99; Table 1, Supplementary-File-Fig. 12).

According to the findings of this meta-analysis, the intervention did not affect the LGA and SGA birth rates. However, in the subgroup analysis based on type of intervention, it was observed that the diet-exercise and diet interventions did not affect the risk of giving birth to an LGA, whereas the exercise intervention significantly lowered the risk (respectively; *z* = 0.73, *p* = 0.47; *z* = 0.78, *p* = 0.44; *z* = 2.19, *p* = 0.03; Table 1, Supplementary-File-Fig. 13). Results for LBW were reported in two studies [27, 28] (*z* = 0.57, *p* = 0.57) and for macrosomia in nine studies [26, 28, 32, 34, 38, 40, 41, 44, 45] (*z* = 1.09, *p* = 0.28) reviewed in this systematic review. The pooled results of these studies showed no differences between the intervention and control groups in terms of these factors (Table 1, Supplementary-File-Fig. 14).

In two studies [27, 32] included in this review, it was found that the diet-exercise and metformin interventions reduced the risk of congenital anomaly, but the difference between the groups was not statistically significant (*z* = 0.43, *p* = 0.67). The results of the diet-exercise and metformin interventions in the subgroup analysis according to the type of intervention were similar to those of the meta-analysis (Table 1, Supplementary-File-Fig. 15).

In the meta-analysis, it was found that weight management interventions addressing pregnant women with obesity had no effect on either admission to the neonatal intensive care unit or on perinatal deaths (respectively; *z* = 1.02, *p* = 0.31; *z* = 0.85, *p* = 0.39). In the subgroup analysis based on the type of intervention, it was found that diet, exercise, and exercise did not affect admissions to the neonatal intensive care unit, but the metformin intervention was a statistically and significantly mitigating factor (*z* = 2.40, *p* = 0.02; Table 1, Supplementary-File-Fig. 15). The pooling of the findings showed that the intervention did not have a statistically significant impact on the development of breastfeeding problems (*z* = 1.21, *p* = 0.22; Table 1, Supplementary-File-Fig. 16).

### Overall Evidence

The degrees of the quality of evidence and the strength of recommendations as assessed by GRADE for all critical results obtained in this systematic review and meta-analysis are presented in Table 2. The certainty of evidence was high in 15 of the outcomes, medium in 13, and low in two.

**Table 2:** GRADE summary of obstetric outcomes of methods used in the management of maternal obesity.

Outcomes	Design/number of studies	№ of Participants		Effects		Certainty of the Evidence [GRADE]	Comments
		Intervention	Control	Relative [95% CI]	Absolute [95% CI]		
Gestational diabetes mellitus	14 RCTs	597/2902 [20.6%]	654/2878 [22.7%]	**OR 0.88** [0.78 to 1.00]	**22 fewerper 1.000** [from 41 fewer to 0 fewer]	⨁⨁⨁⨁ High	Weight loss intervention in maternal obesity does not affect or possibly reduces gestational diabetes.
Gestational hypertension	7 RCTs	60/685 [8.8%]	67/693 [9.7%]	**OR 0.92** [0.64 to 1.33]	**7 fewerper 1.000** [from 33 fewer to 28 more]	⨁⨁⨁⨁ High	Weight loss intervention in maternal obesity does not affect or possibly reduces gestational hypertension.
Pre-eclampsi	6 RCTs	69/1724 [4.0%]	64/1739 [3.7%]	**OR 1.10** [0.78 to 1.56]	**4 more per 1.000** [from 8 fewer to 19 more]	⨁⨁⨁◯ Moderate	
Eclampsia	1 RCTs	6/783 [0.8%]	10/772 [1.3%]	**OR 0.59** [0.21 to 1.63]	**5 fewerper 1.000** [from 10 fewer to 8 more]	⨁⨁◯◯ Low	
Excessive weight gain during pregnancy	3 RCTs	120/383 [31.3%]	107/393 [27.2%]	**OR 1.07** [0.54 to 2.13]	**14 more per 1.000** [from 104 fewer to 171 more]	⨁⨁⨁◯ Moderate	
Abortion	2 RCTs	25/1009 [2.5%]	16/995 [1.6%]	**OR 1.55** [0.82 to 2.93]	**9 more per 1.000** [from 3 fewer to 30 more]	⨁⨁⨁◯ Moderate	
Gestational weight gain	10 RCTs	2206	2218	-	MD **1.24 lower** [1.83 lower to 0.65 lower]	⨁⨁⨁⨁ High	Management of maternal obesity reduces weight gain during pregnancy.
Vaginal delivery	8 RCTs	741/1457 [50.9%]	787/1550 [50.8%]	**OR 1.04** [0.90 to 1.20]	**10 more per 1.000** [from 26 fewer to 45 more]	⨁⨁⨁⨁ High	Weight loss intervention in maternal obesity does not affect or possibly increase the rate of vaginal delivery.
Instrumental delivery	8 RCTs	161/1416 [11.4%]	162/1495 [10.8%]	**OR 1.03** [0.82 to 1.30]	**3 more per 1.000** [from 18 fewer to 28 more]	⨁⨁⨁⨁ High	Weight loss intervention in maternal obesity does not affect or possibly increase the rate of instrumental delivery.
Elective cesarean delivery	6 RCTs	245/1253 [19.6%]	242/1357 [17.8%]	**OR 1.03** [0.71 to 1.50]	**4 more per 1.000** [from 45 fewer to 67 more]	⨁⨁⨁◯ Moderate	
Emergency cesarean delivery	6 RCTs	193/1253 [15.4%]	247/1357 [18.2%]	**OR 0.89** [0.58 to 1.36]	**17 fewerper 1.000** [from 68 fewer to 50 more]	⨁⨁⨁◯ Moderate	
Cesarean delivery	12 RCTs	587/1832 [32.0%]	667/1928 [34.6%]	**OR 0.89** [0.78 to 1.02]	**26 fewerper 1.000** [from 54 fewer to 4 more]	⨁⨁⨁⨁ High	Weight loss intervention in maternal obesity does not affect or possibly reduces cesarean delivery.
Preterm delivery	7 RCTs	31/1381 [2.2%]	29/1388 [2.1%]	**OR 1.10** [0.66 to 1.83]	**2 more per 1.000** [from 7 fewer to 17 more]	⨁⨁⨁⨁ High	
Induction of labour	4 RCTs	321/1013 [31.7%]	339/993 [34.1%]	**OR 0.90** [0.74 to 1.08]	**23 fewerper 1.000** [from 64 fewer to 18 more]	⨁⨁⨁⨁ High	Weight loss intervention in maternal obesity does not affect or possibly reduces induction of labor.
Perineal laceration	1 RCTs	4/18 [22.2%]	2/10 [20.0%]	**OR 1.14** [0.17 to 7.69]	**22 more per 1.000** [from 159 fewer to 458 more]	⨁⨁⨁◯ Moderate	
Large for gestational age	9 RCTs	158/1881 [8.4%]	192/1915 [10.0%]	**OR 0.85** [0.54 to 1.33]	**14 fewerper 1.000** [from 44 fewer to 29 more]	⨁⨁⨁⨁ High	Weight loss intervention in maternal obesity does not affect or possibly reduces the birth of large babies by gestational month.
Small for gestational age	5 RCTs	42/741 [5.7%]	35/736 [4.8%]	**OR 1.20** [0.76 to 1.90]	**9 more per 1.000** [from 11 fewer to 39 more]	⨁⨁⨁⨁ High	Weight loss intervention in maternal obesity does not affect or possibly increase the rate of small for gestational age
Macrosomia	10 RCTs	254/1939 [13.1%]	276/1951 [14.1%]	**OR 0.90** [0.75 to 1.09]	**12 fewerper 1.000** [from 31 fewer to 11 more]	⨁⨁⨁⨁ High	Weight loss intervention in maternal obesity does not affect or possibly reduces the birth of macrosomia
Low birth weight	3 RCTs	30/665 [4.5%]	34/656 [5.2%]	**OR 0.86** [0.52 to 1.43]	**7 fewerper 1.000** [from 24 fewer to 21 more]	⨁⨁⨁⨁ High	Weight loss intervention in maternal obesity does not affect or possibly reduces the birth of Low birth weight
Congenital anomaly	2 RCTs	12/1009 [1.2%]	14/995 [1.4%]	**OR 0.84** [0.39 to 1.84]	**2 fewerper 1.000** [from 9 fewer to 12 more]	⨁⨁⨁◯ Moderate	
Neonatal intensive care unit admission	5 RCTs	89/1136 [7.8%]	105/1143 [9.2%]	**OR 0.86** [0.64 to 1.15]	**12 fewerper 1.000** [from 31 fewer to 12 more]	⨁⨁⨁◯ Moderate	Weight management interventions used during pregnancy may possibly reduce neonatal intensive care unit admission.
Perinatal death	2 RCTs	10/1009 [1.0%]	14/995 [1.4%]	**OR 0.70** [0.31 to 1.59]	**4 fewerper 1.000** [from 10 fewer to 8 more]	⨁⨁⨁◯ Moderate	
Birth weight	14 RCTs	2372	2448	-	MD **51.84 lower** [85.86 lower to 17.83 lower]	⨁⨁⨁⨁ High	Management of maternal obesity reduces birth weight.
Postpartum haemorrhage	2 RCTs	149/1009 [14.8%]	122/995 [12.3%]	**OR 1.24** [0.96 to 1.61]	**25 more per 1.000** [from 4 fewer to 61 more]	⨁⨁⨁◯ Moderate	
reastfeeding problems	2 RCTs	219/592 [37.0%]	311/620 [50.2%]	**OR 0.62** [0.28 to 1.35]	**117 fewerper 1.000** [from 282 fewer to 74 more]	⨁⨁⨁◯ Moderate	

### Publication bias

In findings with a sufficient number of studies ( > 10) reporting the same outcome, funnel plots can be used to screen for publication bias [21]. In this meta-analysis, there were three outcomes that met these criteria, and funnel plots were created for these outcomes. In our study, we adapted existing methods for assessing publication bias in standard systematic reviews by creating a funnel plot for each of the relevant comparisons and overlaying these plots on top of each other while aligning the reference lines. This is called a comparison adjusted funnel plot [20]. There was a small asymmetry in the outcome outcomes of cesarean delivery and gestational diabetes due to the size of the effect, but since these outcomes were homogeneous, it was thought that there was no publication bias. The same category applies to the birth weight outcome, but the group is heterogeneous (Supplementary-File-Fig. 17). To reduce the effect of this outcome, a random effects model was used in the analysis. No obvious asymmetry was observed in the comparison. The reasons for these asymmetries can be explained.

## Discussion

We determined in our study that the exercise program used in the management of maternal obesity statistically significantly reduced the risk of developing gestational diabetes, but that the methods of diet, diet-exercise, leaflet distribution, and metformin administration were relatively ineffective. These findings are consistent with the literature [45, 46]. The findings suggest that the use of exercise interventions in the management of maternal obesity may reduce the development of gestational diabetes.

We found in our study that the methods used in the management of obesity reduced the risk of developing gestational hypertension, but this result was not statistically significant. Similar results were reported by Menichini et al. [45]. However, in the subgroup analysis performed according to the type of intervention in our study, it was determined that the diet exercise intervention had only a borderline effect on reducing the development of gestational hypertension. There are meta-analyses in the literature reporting that different methods have different effects [15, 46, 47]. These results indicate that weight management is necessary in pregnant women with higher body weight; individualized, combined methods will be more effective, and thus complications that may develop due to gestational hypertension can be reduced. It has been reported in the literature that the methods used in the management of obesity do not affect the risk of preeclampsia [45] eclampsia, or abortion [48]. The findings of this study also support the literature. In addition, a recent meta-analysis reported, in contrast to our study, that lifestyle interventions and bariatric surgery reduce the risk of preeclampsia [47] The fact that these results were obtained from the data of a small number of studies indicates that they may be coincidental and that more comprehensive, well designed RCTs are needed on the subject.

We found in our study that the weight loss intervention applied to pregnant women with obesity decreased the weight gained during pregnancy. In the subgroup analyses, leaflet distribution, diet, exercise, and exercise interventions significantly and significantly reduced weight gain during pregnancy, but the use of metformin had no effect. These findings support the existing literature [15, 49] and that health professionals should consider the individual characteristics of pregnant women when selecting the method to apply.

In this meta-analysis, interventions applied to pregnant women with obesity did not statistically affect excess weight gain during pregnancy. In this meta-analysis, interventions applied to pregnant women with higher body weight did not statistically affect excess weight gain during pregnancy. The subgroup analysis also showed that none of the interventions examined affected excessive weight gain during pregnancy. Menichin et al. [45] reported that similarly applied interventions did not prevent excessive weight gain. However, based on the evidence uncovered in a Cochrane study, exercise is of vital importance in preventing excessive weight gain during pregnancy [16]. These findings show that although the methods used in the management of obesity prevent excessive weight gain during pregnancy, they do not reduce it sufficiently according to the Institute of Medicine (IOM) criteria [50]. IOM recommends that pregnant women with obese gain less weight during pregnancy. Although interventions applied before pregnancy have been shown to reduce adverse pregnancy outcomes [47], the fact that similar interventions during pregnancy do not have the same effect is thought to be due to the need for weight gain to be individualized and the excessive weight gain of pregnant women beyond the limits set by the IOM. It is suggested that well-designed further studies are needed to clarify this situation in pregnant women who gain weight according to the IOM guidelines. Moreover, based on this result, it can be concluded that health professionals should identify and implement more effective approaches in this regard.

In this meta-analysis, interventions applied to pregnant women with obesity had no effect on spontaneous vaginal and instrumental delivery. These findings support the literature [46, 49]. The higher rate of vaginal delivery in pregnant women with normal weight [50] shows that performing weight loss interventions before pregnancy may increase the rate of vaginal delivery in women with obesity.

It was determined in this meta-analysis that interventions applied to pregnant women with obesity decreased the rate of cesarean section and that the diet-exercise intervention also brought down the rate of emergency cesarean section, but this was not statistically significant. The findings of our study support the literature [45, 46, 49]. The results that healthcare professionals creating individual weight management intervention programs for pregnant women with obesity may decrease the rates of cesarean delivery and emergency cesarean sections.

Similar to the findings of this study, previous studies have revealed that weight management interventions in pregnant women with maternal obesity do not affect preterm birth [45, 46]. The results of studies and the fact that the rate of preterm birth is lower in normal-weight pregnant women [51, 52] points to how important it is for health professionals to implement weight management interventions before pregnancy to ensure a lower preterm birth rate and to make sure that women are at their ideal weight when they become pregnant.

In this study, it was shown that exercise intervention statistically and significantly reduced the percentage of babies with LGA. In this meta-analysis, interventions applied to pregnant women with obesity did not have an impact on small-for-gestational-age (SGA) births and LBW. Results that are consistent with our study have also been reported in the literature [45, 46, 49]. These results show that weight management interventions are not effective in preventing SGA and LBW. It was further found in this meta-analysis that weight management interventions applied to pregnant women with higher body weight did not affect fetal macrosomia. Our study supports the literature [45]. However, in a study, previous bariatric surgery was reported to have reduced complications, such as macrosomia, in mothers with higher body weight [53]. The differences between these results indicate that more studies are needed on this subject.

In the analysis of this study, we determined that interventions applied to pregnant women with obesity did not have an effect on congenital anomalies, admissions of new-borns to intensive care units, or perinatal death. However, previous studies have reported different findings [12, 45, 49]. This difference may be related to the characteristics of the sample group in the studies that were conducted, as well as the characteristics of the individual interventions; it is clear that there is a need for well-designed studies on these important issues.

The meta-analysis showed that interventions applied to pregnant women with obesity, specifically leaflet distribution and exercise interventions, had a statistically significant impact on reducing birth weight. It has been reported in the literature that diet, exercise, and lifestyle interventions applied to pregnant women with obesity do not affect birth weight [45, 46], but weight loss before pregnancy can in fact reduce birth weight [48]. According to these results, health professionals who provide antenatal care to pregnant women with obesity can use methods such as distributing brochures and implementing exercise programs to reduce the risk of macrosomia.

We determined, in line with the literature [48], that interventions applied to pregnant women with obesity did not affect the risk of postpartum hemorrhage. On the other hand, another study reported that obesity increased the risk of postpartum bleeding [54]. Again, in this study; it was found that the exercise intervention applied to pregnant women with obesity had no effect on perineal laceration. The fact that these results were obtained from a small number of studies that the data is insufficient for conclusions, indicating that more RCTs are needed.

Maternal obesity may cause breastfeeding difficulties [55]. This meta-analysis showed that interventions applied to pregnant women with obesity did not affect the emergence of breastfeeding problems. These results indicate that special counseling on this issue during pregnancy and the postpartum period may increase the success of breastfeeding, especially for women with high pre-pregnancy BMI. This information can help health professionals identify pregnant women who are less likely to breastfeed and target early intervention.

### Strengths and limitations of the study

The strengths of this study are that it is based on extensive screening resources; the studies examined are of RCT design and are up-to-date; most of them were conducted in various developed countries. In addition, the risk of bias was low, and the findings included in the analysis were determined by concrete and measurable methods. The inability to include studies published in languages other than English and Turkish, the fact that meta-analyses for some results are based on studies with small numbers and a small sample size, the high heterogeneity between the studies, and the differences in the month of pregnancy in which the intervention started may be limitations.

## Conclusions

The study revealed that methods used in the treatment of maternal obesity may reduce some negative maternal and newborn outcomes, but it is more important to start pregnancy at an ideal weight. Based on these results, it can be suggested that women’s health service providers should strive for the early detection of maternal obesity before and during pregnancy, implement individual-specific methods, encourage individuals to achieve behavioral change, and evaluate the results of the practice. Raising awareness among health professionals, mothers-to-be, families and the community about the management of obesity and its effects on mother-infant health through the work of health professionals in formal and non-formal education services may benefit the protection and improvement of health. Health administrators can create, implement, and evaluate health policies that can improve the effects of maternal obesity on mother-infant health. In addition, it is recommended that more experimental and meta-analysis studies are conducted with a high level of evidence to enable the determination of individual-specific methods with higher efficiency and applicability in the management of maternal obesity.

## Supplementary information

Supplementary file

## References

[1] The World Health Organization [2022]. Obesity [online]. https://www.who.int/health-topics/obesity#tab=tab_1.

[2] The World Health Organization [2022]. Obesity and overweigh. https://www.who.int/news-room/fact-sheets/detail/obesity-and-overweight.

[3] Dolin CD, Kominiarek MA. Pregnancy in women with obesity. Obstet Gynecol Clin N. Am. 2018;45:217–32. 10.1016/j.ogc.2018.01.005. 10.1016/j.ogc.2018.01.005 29747727

[4] Chen C, Xu X, Yan Y. Estimated global overweight and obesity burden in pregnant women based on panel data model. PLOS ONE. 2018;13:0202183. 10.1371/journal.pone.0202183. 10.1371/journal.pone.0202183 PMC6084991 30092099

[5] Australian Institute of Health and Welfare [AIHW] [2017]. Australia’s mothers and babies. https://www.aihw.gov.au/getmedia/2a0c22a2-ba27-4ba0-ad47-ebbe51854cd6/aihw-per-100-in-brief.pdf.

[6] Bjermo H, Lind S, Rasmussen F. The educational gradient of obesity increases among Swedish pregnant women: a register-based study. BMC. Public Health 2015;15. 10.1186/s12889-015-1624-6. 10.1186/s12889-015-1624-6 PMC4391086 25886465

[7] Lahti-Pulkkinen M, Bhattacharya S, Wild SH, Lindsay RS, Räikkönen K, Norman, JE et al. Consequences of being overweight or obese during pregnancy on diabetes in the offspring: a record linkage study in Aberdeen, Scotland. Diabetologia. 2019;62:1412–19. 10.1007/s00125-019-4891-4. 10.1007/s00125-019-4891-4 PMC6647186 31214738

[8] Onubi OJ, Marais D, Aucott L, Okonofua F, Poobalan AS. Maternal obesity in Africa: a systematic review and meta-analysis. J Public Health. 2015;38:218–31. 10.1093/pubmed/fdv138. 10.1093/pubmed/fdv138 PMC5072166 26487702

[9] Alan Dikmen H, Çankaya S. The effect of maternal obesity on prenatal attachment. AUHSJ. 2018;9:118–23. 10.31067/0.2018.1.

[10] Poston L, Caleyachetty R, Cnattingius S, Corvalán C, Uauy R, Herring S, et al. Preconceptional and maternal obesity: epidemiology and health consequences. Lancet Diabetes Endocrinol. 2016;4:1025–36. 10.1016/s2213-8587[16]30217-024. 27743975 10.1016/S2213-8587(16)30217-0

[11] Kutchi I, Chellammal P, Akila A. Maternal obesity and pregnancy outcome: in perspective of new Asian Indian guidelines. J Obstet Gynaecol India. 2020;70:138–44. 10.1007/s13224-019-01301-8. 32255952 10.1007/s13224-019-01301-8 PMC7109213

[12] Cattane N, Räikkönen K, Anniverno R, Mencacci C, Riva MA, Pariante CM, et al. Depression, obesity and their comorbidity during pregnancy: effects on the offspring’s mental and physical health. Mol Psychiatr. 2020;26:462–81. 10.1038/s41380-020-0813-6. 10.1038/s41380-020-0813-6 PMC7850968 32632208

[13] Dutton H, Borengasser SJ, Gaudet LM, Barbour LA, Keely EJ. Obesity in pregnancy: Optimizing outcomes for mom and baby. Med Clin N. Am. 2018;102:87–106. 10.1016/j.mcna.2017.08.008. 29156189 10.1016/j.mcna.2017.08.008 PMC6016082

[14] The World Health Organization European Regional Obesity Report [2022]. Copenhagen: WHO Regional Office for Europe. Licence: CC BY-NC-SA 3.0 IGO. https://iris.who.int/bitstream/handle/10665/353747/9789289057738-eng.pdf.

[15] Muktabhant B, Lawrie TA, Lumbiganon P, Laopaiboon M. Diet or exercise, or both, for preventing excessive weight gain in pregnancy. Cochrane Database Syst Rev. 2015;15:CD007145. 10.1002/14651858.cd007145.pub.3. 10.1002/14651858.CD007145.pub3 PMC9428894 26068707

[16] Adamo KB, Ferraro ZM, Goldfield G, Keely E, Stacey D, Hadjiyannakis S, et al. The maternal obesity management [mom] trial protocol: A lifestyle intervention during pregnancy to minimize downstream obesity. Contemp Clin Trials. 2013;35:87–96. 10.1016/j.cct.2013.02.010. 23459089 10.1016/j.cct.2013.02.010

[17] Furber CM, McGowan L, Bower P, Kontopantelis E, Quenby S, Lavender T. Antenatal interventions for reducing weight in obese women for improving pregnancy outcome. Cochrane Database Syst Rev. 2013. 10.1002/14651858.cd009334.pub2. 23440836 10.1002/14651858.CD009334.pub2 PMC11297397

[18] Aung W, Saw L, Sweet L. An integrative review of interventions for limiting gestational weight gain in pregnant women who are overweight or obese. Women Birth. 2022;35:108–26. 10.1016/j.wombi.2021.04.009. 33958291 10.1016/j.wombi.2021.04.009

[19] The International Weight Management in Pregnancy [i-WIP] Collaborative Group. Effect of diet and physical activity-based interventions in pregnancy on gestational weight gain and pregnancy outcomes: Meta-analysis of individual participant data from randomised trials. *BMJ*. 2017. j3119. 10.1136/bmj.j3119. 10.1136/bmj.j3119 PMC6887834 28724518

[20] Page MJ, McKenzie JE, Bossuyt PM, Boutron I, Hoffmann TC, Mulrow CD, et al. The PRISMA 2020 statement: an updated guideline for reporting systematic reviews. BMJ. 2021;372:n71. 10.1136/bmj.n71. 33782057 10.1136/bmj.n71 PMC8005924

[21] Higgins JPT, Thomas J, Chandler J, Cumpston M, Li T, Page MJ, et al. (editors). Cochrane Handbook for Systematic Reviews of Interventions. 2nd Edition. Chichester (UK): John Wiley & Sons, 2019. Cochrane 2019. [online]. Website https://training.cochrane.org/handbook#how-to-cite [accessed 12 May 2023].

[22] Sterne, JACSJ, Page MJ, Elbers RG, Elbers RG, Blencowe NS, et al. RoB 2: a revised tool for assessing risk of bias in randomised trials. BMJ. 2019;366:l4898. 31462531 10.1136/bmj.l4898

[23] JBI. Data Extraction Forms. 2022. https://jbi-globalwiki.refined.site/space/MANUAL/4687700 [accessed 12 May 2023].

[24] Bogaerts AF, Devlieger R, Nuyts E, Witters I, Gyselaers W, Van den Bergh BR. Effects of lifestyle intervention in obese pregnant women on gestational weight gain and mental health: a randomized controlled trial. Int J Obes. 2013;37:814–21. 10.1038/ijo.2012.162. 10.1038/ijo.2012.162 23032404

[25] Braeken MAKA, Bogaerts A. Effect of lifestyle interventions in obese pregnant women on the neurocognitive development and anthropometrics of preschool children. Obes Facts. 2020;1:11. 10.11159/000506690. 10.1159/000506690 PMC7250361 32268328

[26] Harreiter J, Simmons D, Desoye G, Corcoy R, Adelantado JM, Devlieger R, et al. Nutritional lifestyle intervention in obese pregnant women, including lower carbohydrate intake, is associated with increased maternal free fatty acids, 3-β-hydroxybutyrate, and fasting glucose concentrations: A secondary factorial analysis of the European multicenter, randomized controlled DALI lifestyle intervention trial. Diabetes Care. 2019;42:1380–89. 10.2337/dc19-0418. 31182492 10.2337/dc19-0418

[27] Chiswick CA, Reynolds RM, Denison FC, Whyte SA, Drake AJ, Newby DE, et al. Effect of metformin on maternal and fetal outcomes in obese pregnant women [EMPOWaR]: a randomised, double-blind, placebo-controlled trial. Lancet Diabetes Endocrinol. 2015;3:778–86. 10.1016/s2213-8587[15]00219-3. 26165398 10.1016/S2213-8587(15)00219-3 PMC4673088

[28] Dalrymple KV, Tydeman FAS, Taylor PD, Flynn AC, O’Keeffe M, Briley AL, et al. Adiposity and cardiovascular outcomes in three-year-old children of participants in UPBEAT, an RCT of a complex intervention in pregnant women with obesity. Pediat Obes. 2020;16. 10.1111/ijpo.12725. 10.1111/ijpo.12725 PMC7116719 32914569

[29] Mills HL, Patel N, White SL, Pasupathy D, Briley AL, Santos Ferreira DL, et al. The effect of a lifestyle intervention in obese pregnant women on gestational metabolic profiles: findings from the UK pregnancies better eating and activity trial [UPBEAT] randomised controlled trial. BMC Med. 2019;17. 10.1186/s12916-018-1248-7. 10.1186/s12916-018-1248-7 PMC6340185 30661507

[30] Patel N, Godfrey KM, Pasupathy D, Levin J, Flynn AC, Hayes L, et al. Infant adiposity following a randomised controlled trial of a behavioural intervention in obese pregnancy. Int J Obes. 2017;41:1018–26. 10.1038/ijo.2017.44140. 10.1038/ijo.2017.44 PMC5482395 28216644

[31] Patel N, Hellmuth C, Uhl O, Godfrey K, Briley A, Welsh P, et al. Cord metabolic profiles in obese pregnant women: insights into offspring growth and body composition. Clin Endocrinol Metab. 2018;103:346–55. 10.1210/jc.2017-00876. 10.1210/jc.2017-00876 PMC5761489 29140440

[32] Poston L, Bell R, Croker H, Godfrey KM, Nelson SM, Oteng-Ntim E, et al. Effect of a behavioural intervention in obese pregnant women [the UPBEAT study]: a multicentre, randomised controlled trial. Lancet Diabetes Endocrinol. 2015;3:767–77. 10.1016/s2213-8587[15]00227-2. 26165396 10.1016/S2213-8587(15)00227-2

[33] Garnæs KK, Mørkved S, Salvesen Ø, Moholdt T. Exercise training and weight gain in obese pregnant women: a randomized controlled trial [ETIP Trial]. PLoS Med. 2016;13:1002079. 10.1371/journal.pmed.1002079. 10.1371/journal.pmed.1002079 PMC4961392 27459375

[34] Garnæs KK, Nyrnes SA, Salvesen KÅ, Salvesen Ø, Mørkved S, Moholdt T. Effect of supervised exercise training during pregnancy on neonatal and maternal outcomes among overweight and obese women. Secondary analyses of the ETIP trial: A randomised controlled trial. PLoS One. 2017;12:0173937. 10.1371/journal.pone.0173937. 10.1371/journal.pone.0173937 PMC5360254 28323893

[35] Nyrnes SA, Garnæs KK, Salvesen Ø, Timilsina AS, Moholdt T, Ingul CB. Cardiac function in newborns of obese women and the effect of exercise during pregnancy. A randomized controlled trial. PLoS One. 2018;13:0197334. 10.1371/journal.pone.0197334. 10.1371/journal.pone.0197334 PMC5983429 29856768

[36] Gesche J, Renault K, Nørgaard K, Nilas L. Representativeness of participants in a lifestyle intervention study in obese pregnant women - the difference between study participants and non-participants. Obes Facts. 2014;7:351–60. 10.1159/000369769. 25428213 10.1159/000369769 PMC5644893

[37] Vinter CA, Jensen DM, Ovesen P, Beck-Nielsen H, Tanvig M, Lamont RF, et al. Postpartum weight retention and breastfeeding among obese women from the randomized controlled Lifestyle in pregnancy [LiP] trial. Acta Obstet Gynecol Scand. 2014;93:794–801. 10.1111/aogs.12429. 24834792 10.1111/aogs.12429

[38] Vinter CA, Tanvig MH, Christensen MH, Ovesen PG, Jørgensen JS, Andersen MS, et al. Lifestyle intervention in danish obese pregnant women with early gestational diabetes mellitus according to WHO 2013 criteria does not change pregnancy outcomes: results from the LiP study. Diabetes Care. 2018;41:2079–85. 10.2337/dc18-0808. 30061318 10.2337/dc18-0808

[39] Simpson SA, Coulman E, Gallagher D, Jewell K, Cohen D, Newcombe RG, et al. Healthy eating and lifestyle in pregnancy [HELP]: a cluster randomised trial to evaluate the effectiveness of a weight management intervention for pregnant women with obesity on weight at 12 months postpartum. Int J Obes. 2021;45:1728–39. 10.1038/s41366-021-00835-0. 10.1038/s41366-021-00835-0 PMC8310786 34021264

[40] Vesco KK, Karanja N, King JC, Gillman MW, Leo MC, Perrin N, et al. Efficacy of a group-based dietary intervention for limiting gestational weight gain among obese women: A randomized trial. Obesity. 2014;22:1989–96. 10.1002/oby.20831. 25164259 10.1002/oby.20831 PMC4407817

[41] Wang C, Wei Y, Zhang X, Zhang Y, Xu Q, Sun Y, et al. A randomized clinical trial of exercise during pregnancy to prevent gestational diabetes mellitus and improve pregnancy outcome in overweight and obese pregnant women. Am J Obstet Gynecol. 2017;216:340–51. 10.1016/j.ajog.2017.01.037. 28161306 10.1016/j.ajog.2017.01.037

[42] Bruno R, Petrella E, Bertarini V, Pedrielli G, Neri I, Facchinetti F. Adherence to a lifestyle programme in overweight/obese pregnant women and effect on gestational diabetes mellitus: a randomized controlled trial. Matern Child Nutr. 2016;13:12333. 10.1111/mcn.12333. 10.1111/mcn.12333 PMC6866030 27647837

[43] Sales WB, Nascimento IBD, Dienstmann G, Souza MLR, Silva GDD, Silva JC. Effectiveness of metformin in the prevention of gestational diabetes mellitus in obese pregnant women. Rev Bras Ginecol Obstet. 2018;40:180–87. 10.1055/s-0038-1642632. 29702716 10.1055/s-0038-1642632 PMC10309478

[44] Poston L, Briley AL, Barr S, Bell R, Croker H, Coxon K, et al. Developing a complex intervention for diet and activity behaviour change in obese pregnant women [the UPBEAT trial]; assessment of behavioural change and process evaluation in a pilot randomised controlled trial. BMC Pregnancy Childbirth. 2013;13:148. 10.1186/1471-2393-13-148. 23855708 10.1186/1471-2393-13-148 PMC3718630

[45] Menichini D, Petrella E, Dipace V, Di Monte A, Neri I, Facchinetti F. The impact of an early lifestyle intervention on pregnancy outcomes in a cohort of insulin-resistant overweight and obese women. Nutrients. 2020;12:1496. 10.3390/nu12051496. 32455565 10.3390/nu12051496 PMC7285042

[46] Du MC, Ouyang YQ, Nie XF, Huang Y, Redding SR. Effects of physical exercise during pregnancy on maternal and infant outcomes in overweight and obese pregnant women: A meta-analysis. Birth. 2018;46:211–21. 10.1111/birt.12396. 30240042 10.1111/birt.12396

[47] Schenkelaars N, Rousian M, Hoek J, Schoenmakers S, Willemsen S, Steegers-Theunissen R. Preconceptional maternal weight loss and hypertensive disorders in pregnancy: A systematic review and meta-analysis. Eur J Clin Nutr. 2021;75:1684–97. 10.1038/41430-021-00902-9. 33837274 10.1038/s41430-021-00902-9

[48] Al-Nimr RI, Hakeem R, Moreschi JM, Gallo S, McDermid JM, Pari-Keener M, et al. Effects of bariatric surgery on maternal and infant outcomes of pregnancy-an evidence analysis center systematic review. J Acad Nutr Diet. 2019;119:1921–43. 10.1016/j.jand.2019.02.008. 31040070 10.1016/j.jand.2019.02.008

[49] Okesene-Gafa KAM, Li M, McKinlay CJD, Taylor RS, Rush EC, Wall CR, et al. Effect of antenatal dietary interventions in maternal obesity on pregnancy weight-gain and birthweight: healthy mums and babies [HUMBA]randomized trial. Am J Obst Gynecol. 2019;221:152.e1/152.e13. 10.1016/j.ajog.2019.03.00. 10.1016/j.ajog.2019.03.003 30878323

[50] Institute of Medicine [IOM]. Wei gestasyonel hypertension gain during pregnancy: reexamining the guidelines. National Academies Press: Washington, DC,2009. 20669500

[51] Melchor I, Burgos J, Del Campo A, Aiartzaguena A, Gutiérrez J, Melchor JC. Effect of maternal obesity on pregnancy outcomes in women delivering singleton babies: a historical cohort study. J Perinat Med. 2019;47:625–30. 10.1515/jpm-2019-0103.gt. 31141492 10.1515/jpm-2019-0103

[52] Denison FC, Aedla NR, Keag O, Hor K, Reynolds RM, Milne A, et al. Care of women with obesity in pregnancy: Green-top guideline No. 72. BJOG. 2018;72:62–106. 10.1111/1471-0528.15386. 10.1111/1471-0528.15386 30465332

[53] Yi X, Li Q, Zhang J, Wang Z. A meta-analysis of maternal and fetal outcomes of pregnancy after bariatric surgery. Int J Gyneco Obstet. 2015;130:3–9. 10.1016/j.ijgo.2015.01.011. 10.1016/j.ijgo.2015.01.011 25863541

[54] Overcash RT, Lacoursıere DY. The clinical approach to obesity in pregnancy. Clin Obstet Gynecol. 2014;57:485–500. 10.1097/grf.0000000000000042. 25022997 10.1097/GRF.0000000000000042

[55] Ramji N, Quinlan J, Murphy P, Crane JMG. The impact of maternal obesity on breastfeeding. J Obstet Gynaecol Can. 2016;38:703–11. 10.1016/j.jogc.2016.03.01. 27638980 10.1016/j.jogc.2016.03.013

