# Supplementary material for: The effect of methods used in the management of maternal obesity on pregnancy and birth outcomes: a systematic review with meta-analysis
Source: Int J Obes (Lond). 2025 Mar 26;49(6):1013–23. doi: 10.1038/s41366-025-01748-y (PMC12158762; doi:10.1038/s41366-025-01748-y)
Supplement: Supplementary file 1 — Supplementary file [file 41366_2025_1748_MOESM1_ESM.docx]

**SUPPLEMENTARY FILE LEGENDS**

**FIGURE 1**. Meta-analysis findings on the development of gestational diabetes mellitus in the intervention and control groups

**FIGURE 2.** Meta-analysis findings on gestational hypertension in intervention and control groups

**FIGURE 3.** Meta-analysis findings on pre-eclampsi/eclampsi in intervention and control groups

**FIGURE 4.** Meta-analysis findings on abortion in intervention and control groups

**FIGURE 5.** Meta-analysis findings on gestational weight gain in the intervention and control groups

**FIGURE 6.** Meta-analysis findings on vaginal delivery in the intervention and control groups

**FIGURE 7.** Meta-analysis findings on cesarean section types in intervention and control groups

**FIGURE 8.** Meta-analysis findings on caesarean delivery in the intervention and control groups

**FIGURE 9.** Meta-analysis findings on induction of labor in the intervention and control groups

**FIGURE 10.** Meta-analysis findings on preterm delivery in the intervention and control groups

**FIGURE 11.** Meta-analysis findings on postpartum maternal health in the intervention and control groups

**FIGURE12.** Meta-analysis findings on birth weight in the intervention and control groups

**FIGURE 13.** Meta-analysis findings regarding the baby's weight for gestational age in the intervention and control groups

**FIGURE 14.** Meta-analysis findings on low birth weight and macrosomia in intervention and control groups

**FIGURE 15.** Meta-analysis findings on neonatal health in the intervention and control group

**FIGURE 16.** Meta-analysis findings on breastfeeding in the intervention and control groups

**FIGURE 17.** Publication Bias Regarding Effects of Diet, Exercise, The Distribution of Brochures, and Metformin on The Outcomes of Pregnancy and Delivery in The Management of Maternal Obesity.

**TABLE 1.** Characteristics of the studies included in the systematic review and meta-analyses

**Supplementary file**


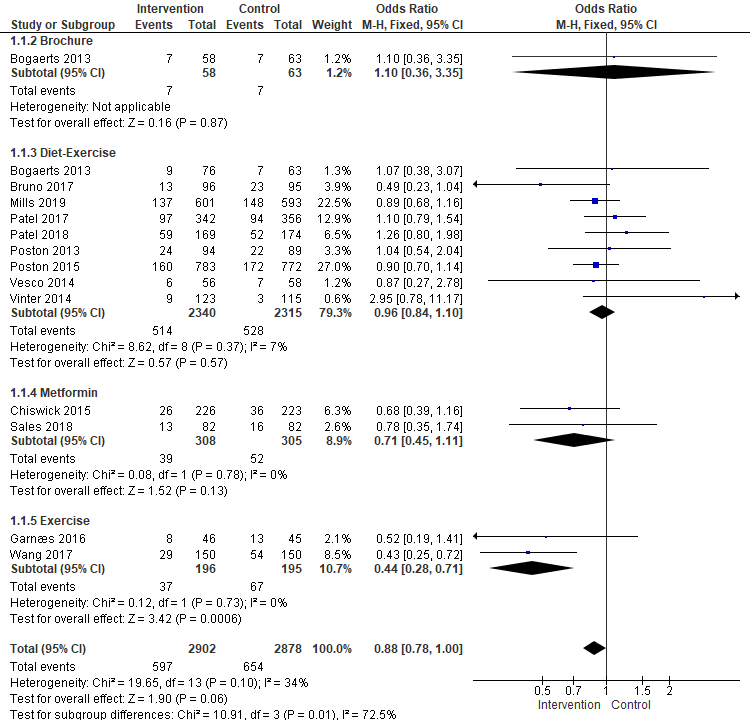


**FIGURE 1.** Meta-analysis findings on the development of gestational diabetes mellitus in the intervention and control groups


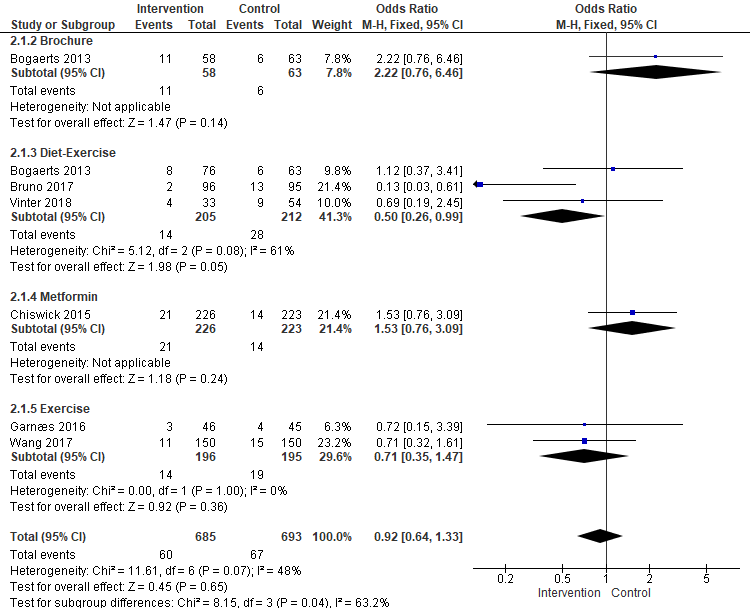


**FIGURE 2.** Meta-analysis findings on gestational hypertension in intervention and control groups

1. Pre-eclampsi
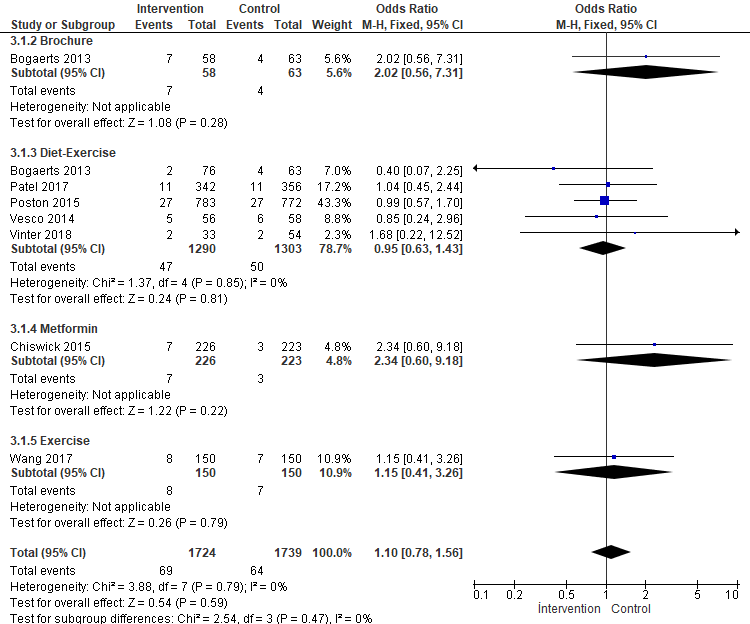

2. Eclampsia


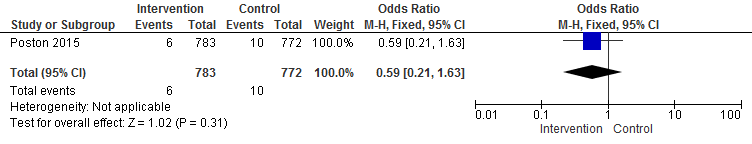


**FIGURE 3.** Meta-analysis findings on pre-eclampsi/eclampsi in intervention and control groups

a) Abortion


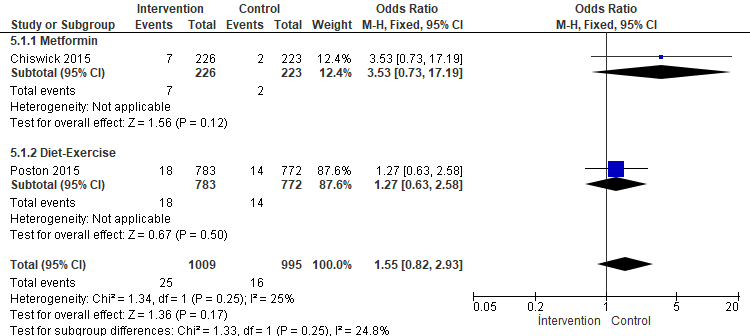


**FIGURE 4.** Meta-analysis findings on abortion in intervention and control groups

1. Gestational weight gain (kg)
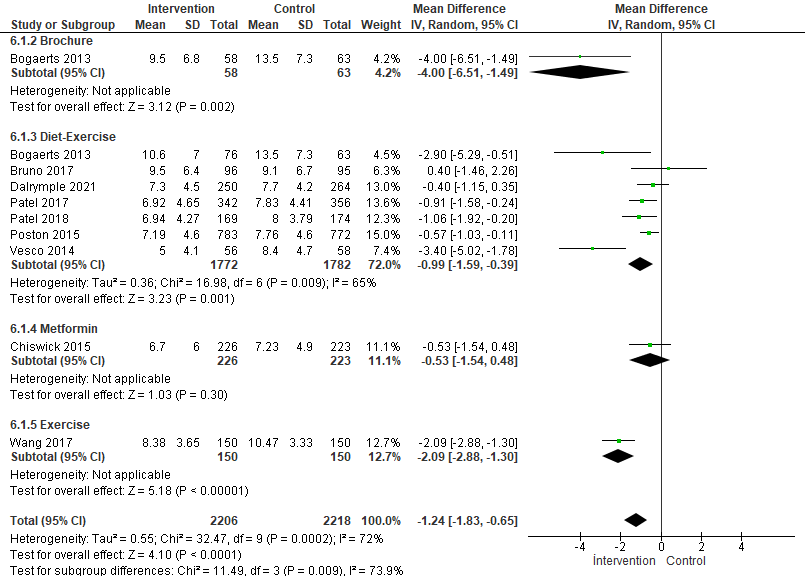

2. Excessive weight gain during pregnancy


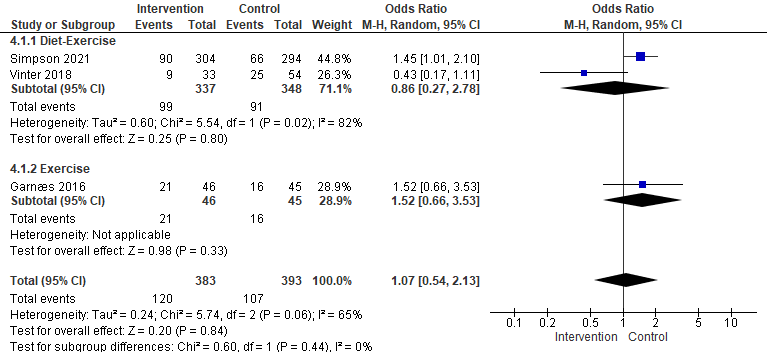


**FIGURE 5.** Meta-analysis findings on gestational weight gain in the intervention and control groups

1. Vaginal delivery


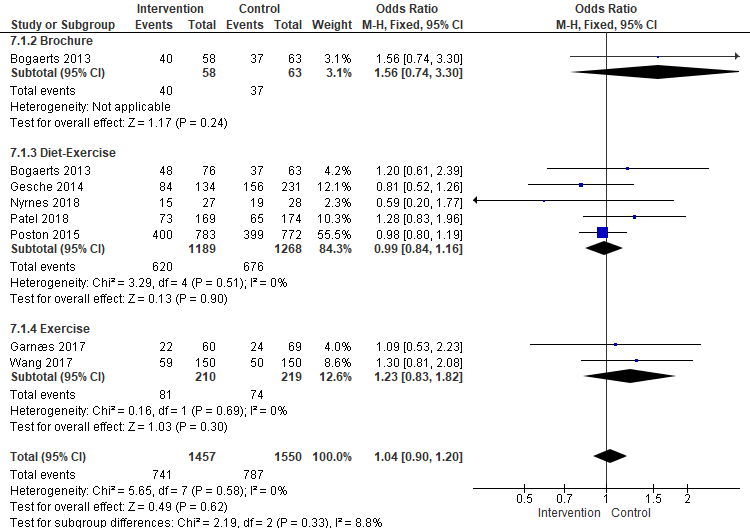


1. Instrumental delivery


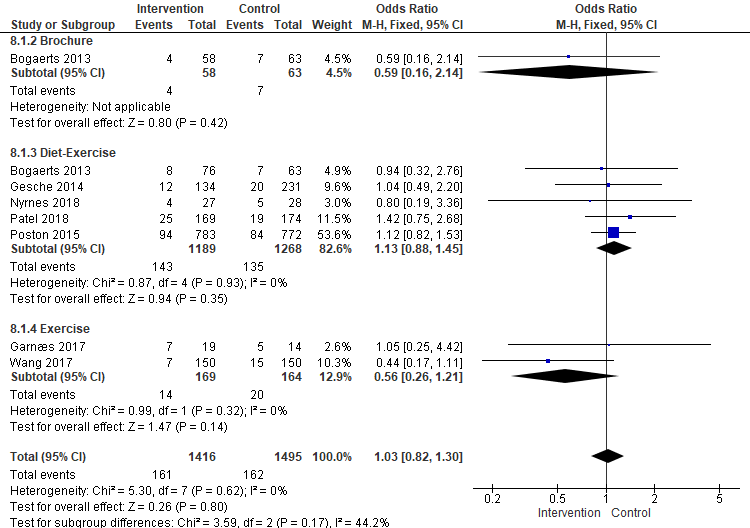


**FIGURE 6.** Meta-analysis findings on vaginal delivery in the intervention and control groups

1. Elective cesarean delivery


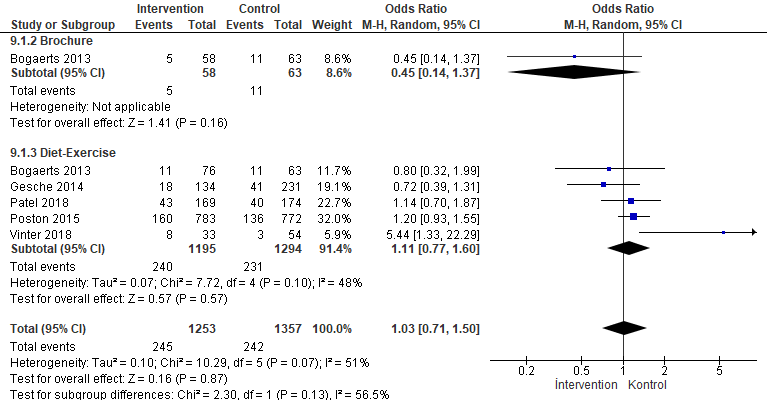


1. Emergency cesarean delivery


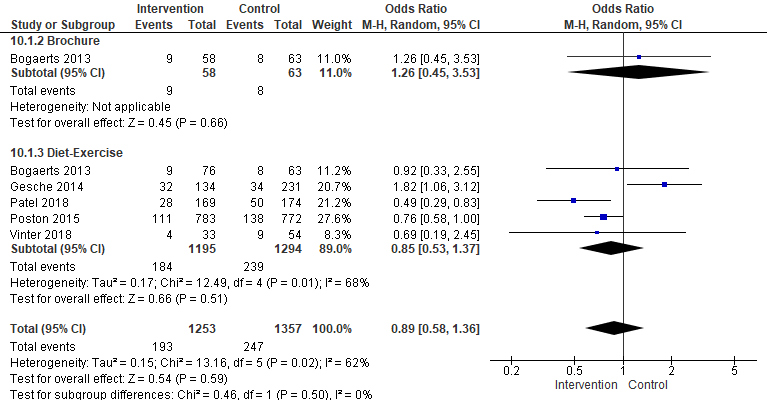


**FIGURE 7.** Meta-analysis findings on cesarean section types in intervention and control groups


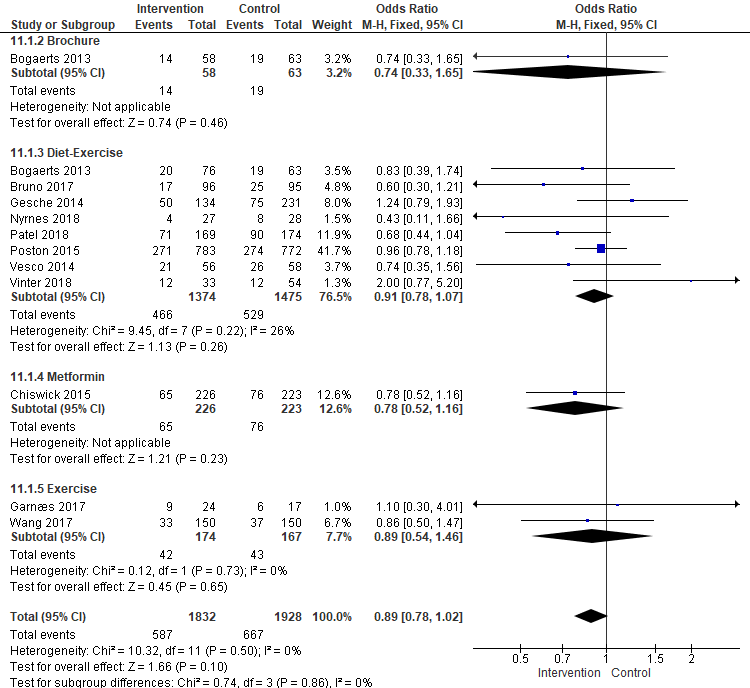


**FIGURE 8.** Meta-analysis findings on caesarean delivery in the intervention and control groups


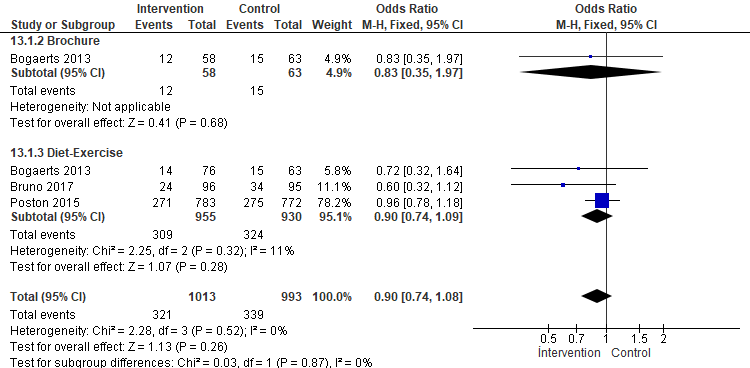


**FIGURE 9.** Meta-analysis findings on induction of labor in the intervention and control groups


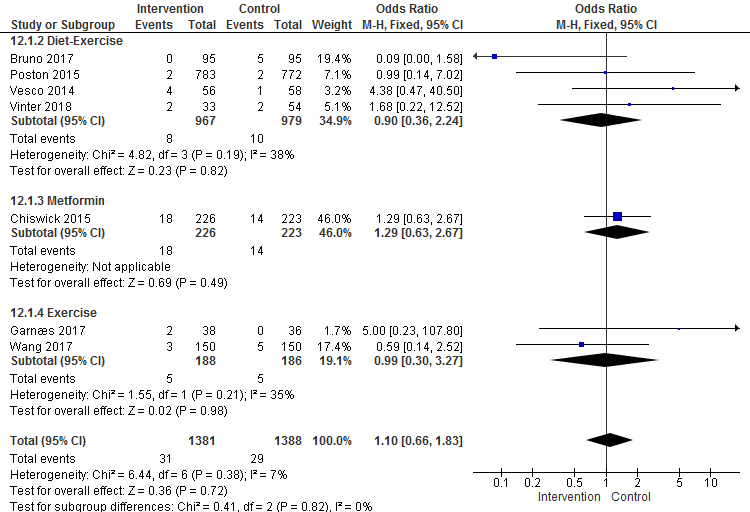


**FIGURE 10.** Meta-analysis findings on preterm delivery in the intervention and control groups

1. Postpartum haemorrhage


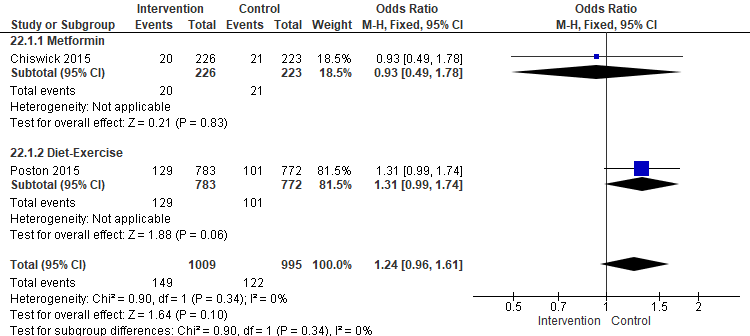


1. 3.4. degree perineal laceration


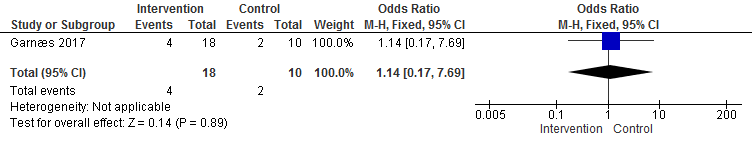


**FIGURE 11.** Meta-analysis findings on postpartum maternal health in the intervention and control groups


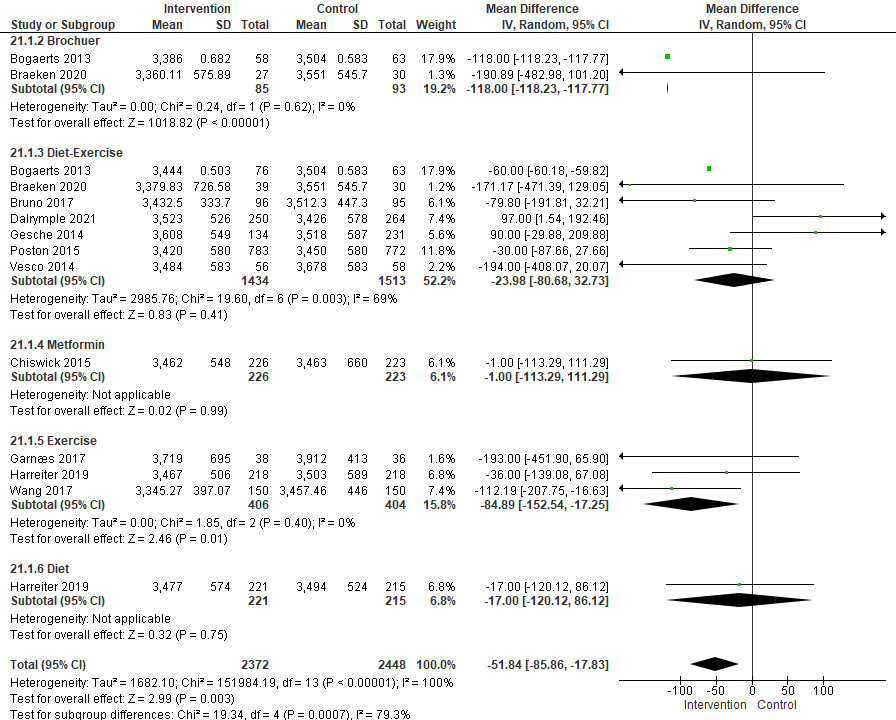


**FIGURE12.** Meta-analysis findings on birth weight in the intervention and control groups

1. Large for gestational age


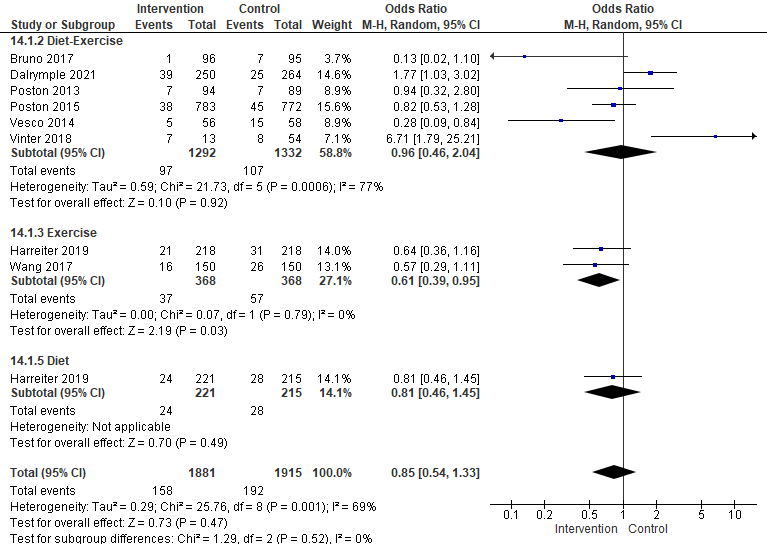


b) Small for gestational age


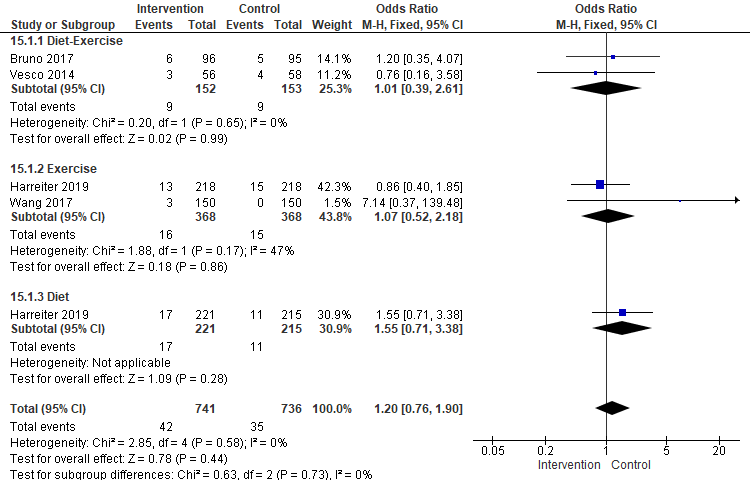


**FIGURE 13.** Meta-analysis findings regarding the baby's weight for gestational age in the intervention and control groups

a) Low birth weight


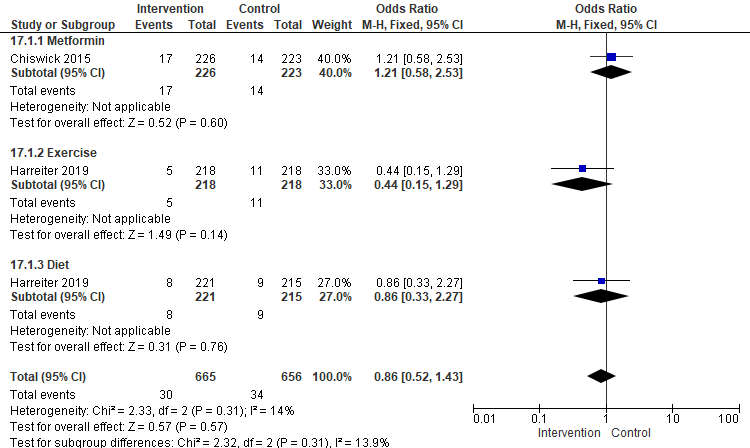


b) Makrozomi


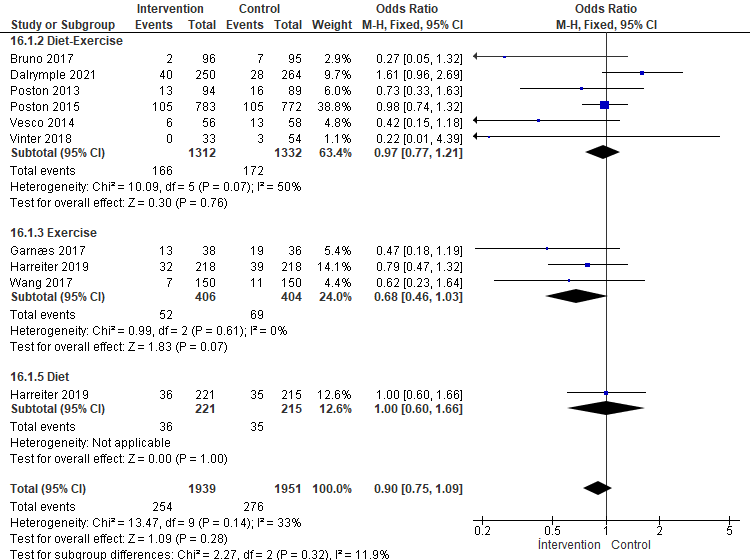


**FIGURE 14.** Meta-analysis findings on low birth weight and macrosomia in intervention and control groups

1. Congenital anomaly


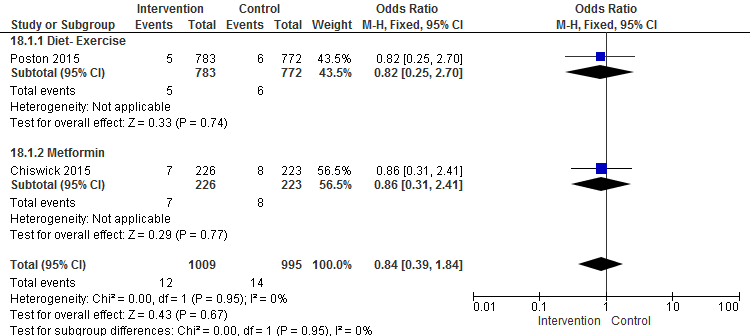


1. Neonatal intensive care unit admission


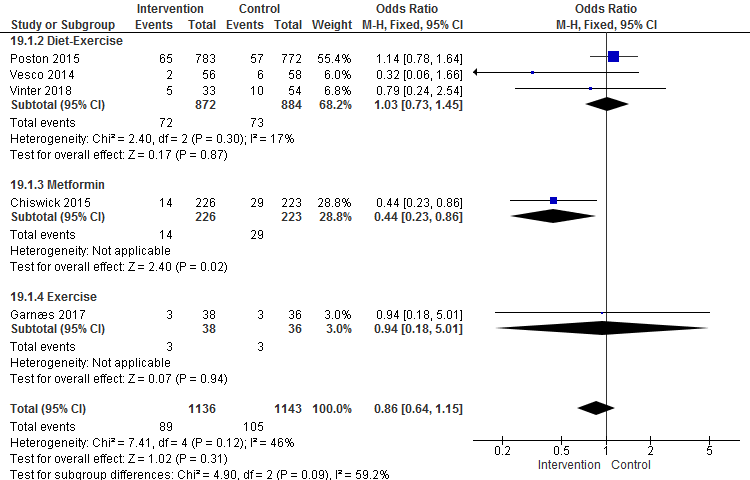


1. Perinatal death


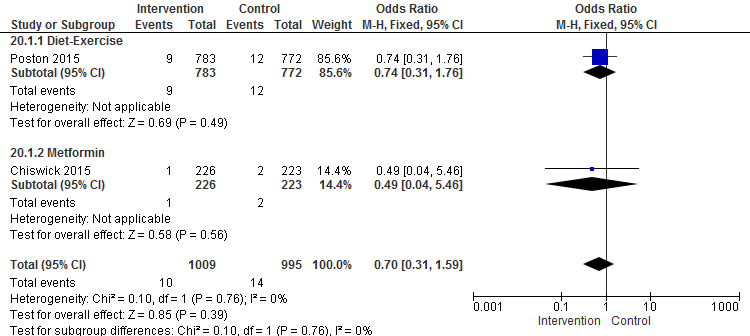


**FIGURE 15.** Meta-analysis findings on neonatal health in the intervention and control group


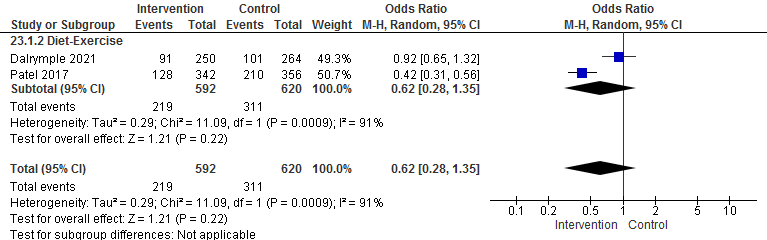


**FIGURE 16.** Meta-analysis findings on breastfeeding in the intervention and control groups

| a) Funnel plot of comparison: Caesarean delivery | b) Funnel plot of comparison: Gestational diabetes mellitus |
| --- | --- |
| 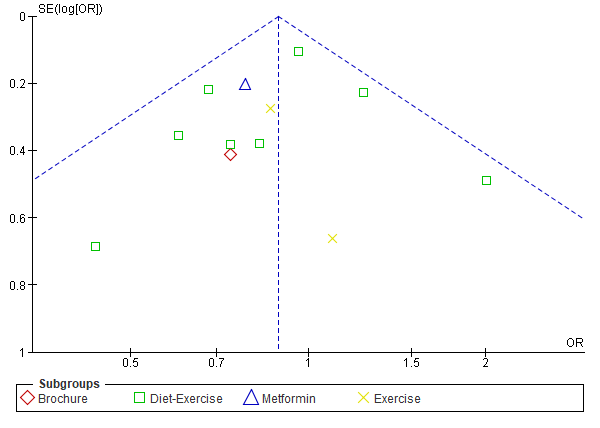 | 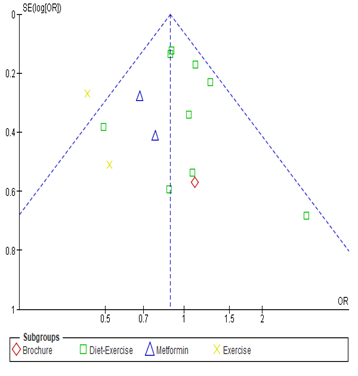 |
| c) Funnel plot of comparison: Birth Weight |  |
| 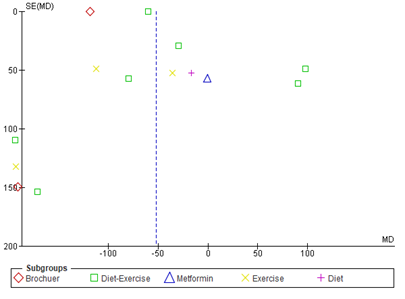 |  |

**FIGURE 17.** Publication Bias Regarding Effects of Diet, Exercise, The Distribution of Brochures, and Metformin on The Outcomes of Pregnancy and Delivery in The Management of Maternal Obesity.

**TABLE 1.** Characteristics of the studies included in the systematic review and meta-analyses

| Author and year/Country | Year of study | Sample size  (Intervention / Control) | BMI  (Intervention/ Control) | Maternal age (Intervention/ Control) | Type of intervention | Data of study |
| --- | --- | --- | --- | --- | --- | --- |
| Bogaerts et al.  2013 / Belgium | 2008 - 2011 | 58 / 76 / 63 | 35.4 ± 5.2^1^  34.4 ± 4.6^2^  34.4 ± 4.1 | 29.6 ± 4.9^1^  28.8 ± 4.5^2^  28.7 ± 4.2 | Brochure^1^  Diet-Exercise^2^ | 1, 2, 3, 4, 5, 6, 7 |
| Braeken and Bogaerts, 2020 / Belgium | 2008 -2011 | 27 / 39 / 30 | 33.09 ± 7.34^1^  34.94 ± 5.25^2^  36.41 ± 5.44 | 29.89 ± 4.19^1^  29.89 ± 4.19^2^  28.67 ± 3.68 | Brochure^1^  Diet-Exercise^2^ | 2 |
| Bruno et al.  2017 / Italy | 2013 -2014 | 96 / 95 | 33.3 ± 6  33.4 ± 5.5 | 31.5 ± 5.0  30.8 ± 5.5 | Diet-Exercise | 1, 2, 3, 4, 6, 7, 8 |
| Chiswick et al.  2015 / England | 2011 -2014 | 226 / 223 | 37.8 ± 4.9  37.7 ± 5.6 | 28.7 ± 5.8  28.9 ± 5.1 | Metformin | 1, 2, 3, 4, 5, 7, 8, 9, 10, 11, 12, 13, |
| Dalrymple et al.  2021 / England | 2014 -2017 | 250 / 264 | 34.5 (32.5 - 38.0)  34.9 (32.6 - 37.8) | 31.2 ± 5.0  31.3 ± 5.5 | Diet-Exercise | 1, 2, 8, 14, 15 |
| Garnæs et al.  2016 / Norway | 2010 -2015 | 46 / 45 | 33.9 ± 3.8 35.1 ± 4.6 | 31.3 ± 3.8  31.4 ± 4.7 | Exercise | 3, 4, 16 |
| Garnæs et al.  2017 / Norway | 2010 -2015 | 38 / 36 | 33.9 ± 3.8 35.1 ± 4.6 | 31.3 ± 3.8  31.4 ± 4.7 | Exercise | 2, 7, 9, 11, 17 |
| Gesche et al.  2014 / Denmark | 2009 -2012 | 134 / 231 | 34.1 ± 4.0  33.9 ± 3.8 | 31.1 ± 4.5  29.9 ± 4.9 | Diet-Exercise | 2, 7 |
| Harreiter et al.  2019 / England | 2012 -2014 | 221 / 215  218 / 18 | 33.8 ± 4.2^1^  33.6 ± 3.8  33.7 ± 3.9^2^  33.7 ± 4.0 | 32.2 ± 5.4^1^  31.7 ± 5.3  31.8 ± 5.2^2^  32.1 ± 5.5 | Diet^1^  Exercise^2^ | Diet; 2  Exercise; 2 |
| Mills et al.  2019 / England | 2015 | 601 / 593 |  |  | Diet-Exercise | 3 |
| Nyrnes et al.  2018 / Norway | 2010 -2015 | 27 / 28 | 33.4 ± 3.4  34.9 ± 3.9 | 31.1 ± 3.0  31.3 ± 4.6 | Exercise | 7 |
| Patel et al.  2017 / England | 2010 -2015 | 342 / 356 | 36.17 ± 4.98  36.31 ± 4.69 | 31.30 ± 5.04  31.00 ± 5.58 | Diet-Exercise | 1, 3, 5, 15 |
| Patel et al.  2018 / England | 2010 -2015 | 169 / 174 | 35.5 (33.0 - 39.1)  35.7 (33.0 - 38.5) | 31.0 (28 - 35)  31.0 (27 - 35) | Diet-Exercise | 1, 3, 7 |
| Poston et al. 2013 / England-Scotland | 2010 -2011 | 94 / 89 | 36.5 ± 4.7  36.1 ± 4.8 | 30.4 ± 5.7  30.7 ± 4.9 | Diet-Exercise | 2, 3 |
| Poston et al.  2015 / England | 2009 -2014 | 783 / 772 | 36.3 ± 4.6  36.3 ± 5.0 | 30.4 ± 5.6  30.5 ± 5.5 | Diet-Exercise | 1, 2, 5, 6, 7, 8, 9, 11, 12, 13, 18 |
| Sales et al. 2018 / Brazil | 2014 -2016 | 82 / 82 | 37.5 ± 4.7  37.5 ± 5.0 | 28.8 ± 6.0  29.7 ± 6.3 | Metformin | 3 |
| Simpson et al. 2021 / USA | 2011-2014 | 304 / 304 | 37.9 ± 5.7  36.5 ± 4.9 | 29.1 ± 5.1  28.8 ± 5.5 | Diet-Exercise | 16 |
| Vesco et al.  2014 / USA | 2009 -2011 | 56 / 58 | 36.8 ± 4.7  36.7 ± 5.2 | 31.2 ± 4.6  32.4 ± 5.1 | Diet-Exercise | 1, 2, 3, 5, 7, 8, 11 |
| Vinter et al.  2014 / Denmark | 2007 -2010 | 123 / 115 | ≥ 30 kg/m ^2^ |  | Diet-Exercise | 3 |
| Vinter et al.  2018 / Denmark | 2010 | 33 / 54 | 34.3 (32.3 - 39.2)  34.6 (32.7 - 37.3) | 29 (27 - 34)  30 (27 - 32) | Diet-Exercise | 2, 4, 5, 7, 8, 11, 16 |
| Wang et al.  2017 / China | 2014 -2016 | 150 / 150 | 39.0 ± 26.0  38.0 ± 25.3 | 32.14 ± 4.57  32.50 ± 4.91 | Exercise | 1, 2, 3, 4, 5, 7, 8 |

1. Gestational weight gain (kg), 2. Newborn weight outcomes, 3. Gestational diabetes mellitus, 4. Gestational hypertension, 5. Pre-eclampsi, 6. Induction of labour, 7. Mode of delivery, 8. Preterm delivery, 9. Postpartum haemorrhage, 10. Abortion, 11. Neonatal intensive care unit admission, 12. Congenital anomaly, 13. Perinatal death, 14. Macrosomia, 15. Breastfeeding problems, 16. Gaining more weight than IOM recommendations during pregnancy, 17. 3.-4. degree perineal laceration, 18. Eclampsia,
